# Physical activity enhances fecal lactobacilli in rats chronically drinking sweetened cola beverage

**DOI:** 10.1515/biol-2022-0070

**Published:** 2022-06-27

**Authors:** Margareta Marusakova, Boris Dudik, Katarina Hadova, Zuzana Kmecova, Eva Kralova, Peter Krenek, Andrea Bilkova, Jan Klimas

**Affiliations:** Department of Pharmacology and Toxicology, Comenius University Bratislava, Bratislava, Slovakia; Department of Cell and Molecular Biology of Drugs, Comenius University Bratislava, Bratislava, Slovakia

**Keywords:** overweight, sweetened beverage, physical activity, fecal *Lactobacillus* abundance, adiponectin

## Abstract

Overweight and obesity have been linked with increased intake of sugar-sweetened beverages. On the other hand, physical activity has been known to lead to weight loss. Therefore, we hypothesized that exercise might influence the *Lactobacillus* population in fecal microbiota as their changed abundance is often associated with shifts in the physical activity and diet. In our experiment, Wistar rats were allocated into groups with normal feed or added sugar-sweetened beverages with or without access to a running wheel. Interestingly, only a combination of physical activity and sweetened beverage intake was associated with a significant increase in fecal lactobacilli abundance, suggesting a connection between exercise and a rise in lactobacilli abundance. Moreover, physical activity has improved weight-related parameters and led to increased plasma and mRNA adiponectin levels. Ghrelin and leptin plasma levels were unaltered. Taken together, our results demonstrate that effect of physical activity on adiposity even during unhealthy feeding patterns is accompanied by increased lactobacilli abundance in the fecal microbiota population.

## 1 Introduction

Consumption of fructose and/or sucrose via ingestion of sugar-sweetened beverages has increased significantly in the last decades, and this overconsumption has been linked with dyslipidemia, obesity, and diabetes epidemic [1]. Low doses of fructose in the diet are metabolized by the small intestine, but at high doses, fructose is metabolized and digested directly by the liver and microbiota, causing fatty liver and obesity [2]. In addition, it has been shown to affect lipid metabolism by increasing plasma triglycerides (TAG) and fasting plasma free fatty acids [3]. Multiple studies suggest a relation between fructose consumption and the onset of obesity and dyslipidemia [4] and often nonalcoholic fatty liver disease [5]. Importantly, sweetened cola beverages’ intake in rodents has been used as an experimental model for studying metabolic syndrome-related features by stimulating the human unhealthy feeding pattern [6,7].

While it is clear that an imbalance between energy intake and energy expenditure leads to body weight gain, it has not been fully explained yet which factor of these two has been more markedly promoting the obesity epidemic [8]. A longitudinal study tracking voluntary exercise in free time and body weight increase in children reported that higher physical activity levels predicted lower adiposity during growth [9]. Similar studies in animals demonstrated that spontaneous physical activity also significantly attenuates adiposity gain [10]. Exercise has a strong positive effect on weight loss in general and insulin sensitization, blood pressure normalization, and overall cardiovascular health [11].

It is well established that the amount and nutrient content of ingested food can affect the microbial composition of the gut; obesity has been linked with modified bacterial diversity as an effect of both high fat and high sugar diets [12]. More recently, an experimental study has indicated that physical activity could alter the gut microbiota in high fat diet-induced obese mice [13], and also, several clinical studies suggested that exercise could play an important role in modifying microbial composition in obese and diabetic individuals [14,15]. Particularly, a varied abundance of the fecal microbiota populations of bacteria from the genus *Lactobacillus* is often associated with changes in physical activity and diet [16]. Although lactobacilli represent only <1% of the total bacterial population in the gut [17], their altered abundance has been related to various metabolic disorders, including type 1 and type 2 diabetes mellitus [18,19]. Relevant to our study, there are conflicting reports on the association of intestinal lactobacilli abundance with obesity in humans [20,21]. Because lactobacilli appear to be associated with both weight increase and weight loss [22], these findings may reflect discrepancies in the lactobacilli properties induced by species and strain differences [23]. Probiotic supplementation of lactobacilli is often associated with a positive influence on a whole-body metabolism by affecting energy balance and inflammation [24], as well as on performance in athletes [25,26], but this effect is also known to be strain specific.

The aim of this study is to investigate the effect of sweetened beverage intake on the overall metabolic state. We hypothesized that physical activity, even during unhealthy feeding represented by sugar-sweetened beverages intake, may attenuate weight gain and obesity-related features in young rats by improving overall metabolic state due to increased energy output and by affecting microbiota composition. We also aim to demonstrate the role of microbiota in obesity prevention in connection with physical exercise.

## 2 Materials and methods

### 2.1 Animal model

Four-week-old male Wistar rats (obtained from a breeding station Dobra Voda, Institute of Experimental Pharmacology & Toxicology, Centre of Experimental Medicine, Slovak Academy of Sciences) were randomly allocated into the following groups: animals drinking tap water (CON, *n* = 10) and animals drinking sweetened cola beverage (Coca-Cola Original^®^) (SSB, *n* = 10) ad libitum, which were further divided into subgroups with access to physical activity [control group drinking water and exercising = SPA (*n* = 10) and group drinking sugar-sweetened beverage and exercising = SSB + SPA (*n* = 10)]. Animals in these subgroups had a running wheel available in their individual cages to allow for spontaneous physical activity. To measure the covered distance, the wheels were equipped with a magnet and a sensor, and upon their passage, the distance was continuously recorded. Randomization into all groups was performed so that the bodyweight would not differ at the beginning of the experiment, and animals were co-housed in groups of 2–3 animals.

Animals had free access to water or decarbonized sweetened cola beverage (according to the manufacturer, 100 mL of this beverage contains 27 g sugars, mainly high fructose corn syrup), were fed a normal chow diet, and the experimental period lasted 6 months. Weight gain and food and liquid intake were recorded weekly. Total caloric intake was calculated as a sum of ingested liquids and solid food for 24 h in a metabolic cage, where individual animals were placed at the end of the experiment. Then, TAG levels and random and fasting glycemia in capillary blood were measured using a commercially available analyzer (Accutrend, Roche, Switzerland). Animals were sacrificed by exsanguination in full anesthesia induced by tribromethanol (dose 13 mL/kg in the form of Avertin solution, Sigma-Aldrich, Germany).


**Ethical approval:** The research related to animal use has been complied with all the relevant national regulations and institutional policies for the care and use of animals, has been conformed to the Guide for the Care and Use of Laboratory Animals (8th edition, National Academies Press) and to the Guide for the Care and Use of Laboratory Animals published in the Collection of Laws of the Slovak Republic, and was approved by the Ethics Committee of the Faculty of Pharmacy, Comenius University and by the State Veterinary and Food Administration of the Slovak Republic.

### 2.2 Origin and cultivation of bacterial strain

*Lactobacillus reuteri* CCM 3625 was purchased from the Czech Collection of Microorganisms (Brno, Czech Republic). The bacterial strain was cultivated in MRS broth (VWR, USA) at 37°C in anaerobic conditions for 18 h.

### 2.3 DNA isolation from fecal samples

Fecal samples harvested on the day of sacrifice individually from each animal were frozen immediately and stored at −80°C until further analysis. Total genomic DNA from feces was isolated by FastDNATM Kit for Feces (mpbio, USA) according to manufacturer’s instructions. The quality and concentration of isolated DNA were verified by spectrophotometry on Epoch microplate spectrophotometer (Biotek, USA), and samples were stored at −20°C until qPCR analysis.

### 2.4 Relative abundance of target bacteria in feces analyzed by qPCR

To quantify the relative abundance of bacteria of genus *Lactobacillus* in feces, qPCR using specific primers for genus *Lactobacillus* (LAC) and universal bacterial primers (UNI) designed on the basis of 16S rRNA gene sequences (Table 1) was conducted. Expressions of studied genes were quantified by qPCR using thermocycler QuantStudioTM 3 (Applied Biosystems, Thermo Fisher Scientific, USA) using HOT FIREPol EvaGreen qPCR mix Plus (Solis, BioDyne, Estonia). The PCR program consisted of initial denaturation at 95°C for 15 min, followed by 40 cycles of 95°C for 15 s, 60°C for 30 s, and 72°C for 30 s. All experiments were conducted in triplicates along with no template control. PCR products were evaluated by melting curve analysis and gel electrophoresis to confirm specific amplification. Amplification efficiency of primers was determined by making serial dilutions of reference bacterial DNA (*L. reuteri* CCM 3625), calculating a linear regression based on the Ct data points, and inferring the efficiency from the slope of the line. qPCR was performed as described above with six 10-fold dilutions of *L. reuteri* CCM 3625 genomic DNA (extracted from 109 CFU using the above-mentioned kit) for both primer pairs. The Ct values obtained from each sample were transformed into a percentage with the equation:

X=\frac{{({\rm{Eff}}\left.{\rm{UNI}})}^{{\rm{Ct\; UNI}}}}{({{\rm{Eff}}\left.{\rm{LAC}})}^{{\rm{Ct\; LAC}}}}\times 100.]

**Table 1:** Primer sequences for bacterial studies and rat tissue qPCR

Target gene	Primer sequence (5′ → 3′)	qPCR efficiency (%)	Reference
Universal bacterial group	F-TCCTACGGGAGGCAGCAGT	96.20	[41]
	R-GGACTACCAGGGTATCTAATCCTGTT		
Lactobacillus genus	F-GAGGCAGCAGTAGGGAATCTTC	99.26	[42]
	R-GGCCAGTTACTACCTCTATCCTTCTTC		
B2m	F-ATGGAGCTCTGAATCATCTGG	NA	This study
	R-AGAAGATGGTGTGCTCATTGC		
Actb	F-CCGCGAGTACAACCTTGTTG	NA	This study
	R-GCAGCGATATCGTCATCCA		
TNF	F-AACTTCGGGGTGATCGGTCCCA	NA	This study
	R-TACGACGTGGGCTACGGGCTT		
Lep	F-AGACCCCAGCGAGGAAAATG	NA	This study
	R-TACCGACTGCGTGTGTGAAA		
GLUT4	F-GACCCGCCCTTTGCACACCA	NA	This study
	R-TCACTCGCTGCTGGGGGGT		
Adiponectin	F-GGGAGACGCAGGTGTTCTTG	NA	This study
	R-CCTACGCTGAATGCTGAGTGA		

Eff. UNI is the calculated efficiency of the universal primers (2 = 100% and 1 = 0%) and Eff. LAC refers to the efficiency of the primers for the genus Lactobacillus. The Cts are the threshold cycles registered by the thermocycler. Resolving this formula, *X* represents the percentage of 16S rRNA taxon-specific copy numbers existing in a sample [27].

### 2.5 RNA isolation and qPCR analysis of adipose tissue

Samples of white adipose tissue were dissected and stored at −80°C until RNA isolation with Tri-Reagent^®^ (Sigma-Aldrich, USA). Afterward, a transcription into cDNA was performed (High capacity cDNA Reverse Transcription Kit with RNAse inhibitor, Applied Biosystems, USA). Complementary DNA was then used for qPCR analysis (StepOne Plus System, Applied Biosystems) using SYBR Select Master Mix (Applied Biosystems) under the following conditions: denaturation at 95°C, annealing at 60°C, and extension at 72°C for 40 cycles. B2-microglobulin (B2m) and beta-actin (Actb) were used as references. We used sequences of primers shown in Table 1. PCR efficiency was calculated by LinReg, the exact percentage is not reported for specific genes. Relative quantification of mRNA expression in the qPCR reaction was calculated by the 2ΔΔCt method.

### 2.6 Plasma concentrations of adiponectin, insulin, and ghrelin

To measure and analyze adiponectin (as an adipose tissue-related indicator of metabolic changes), insulin (as a pancreas-related indicator of metabolic changes), and ghrelin (as a gastrointestinal tract-related indicator of appetite), commercially available kits for ELISA were used (Rat Ghrelin ELISA Kit, Elabscience, USA; Rat Adiponectin ELISA Kit, Abcam, UK; and Rat Insulin ELISA Kit, Mercodia, Sweden, respectively).

### 2.7 Statistical analysis

Results are shown as an average of the whole group ± SEM. Statistical analyses were performed using GraphPad software via one-way analysis of variance with the Bonferroni post hoc test. For *Lactobacillus* studies, Kruskal–Wallis test with Dunn´s multiple comparison test was used. *p* values <0.05 were considered statistically significant.

## 3 Results

### 3.1 Percentage abundance of genus *Lactobacillus* in stool samples

**Figure 1:**
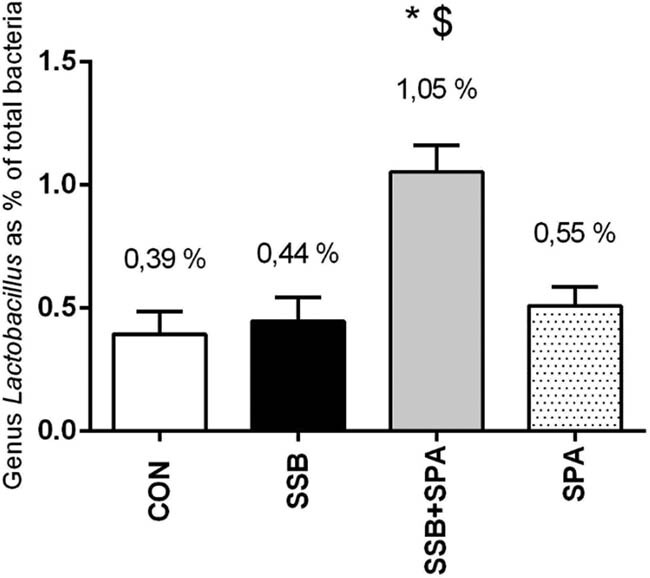
Relative abundance of genus *Lactobacillus* in feces of rats. CON = control group, SSB = group drinking sugar sweetened beverage, SSB + SPA = group drinking sugar sweetened beverage concomitant to spontaneous physical activity, SPA = group performing spontaneous physical activity. Results shown as average ± SEM. *n* = 8–10 per group; *p < 0.01 vs SSB, ^$^ *p* < 0.01 vs SPA.

We observed a significantly higher abundance of genus *Lactobacillus* in feces of the group drinking sweetened beverage while performing physical activity (SSB + SPA, 1.05% of total bacterial population in stool, *n* = 8) vs sedentary group drinking sweetened beverage (SSB, 0.44% of total bacteria, *n* = 9; *p* < 0.05) (Figure 1). Unhealthy feeding (SSB) or physical activity (SPA) alone did not affect the population of lactobacilli.

### 3.2 Effect of physical activity on weight gain and overweight-related features

**Figure 2:**
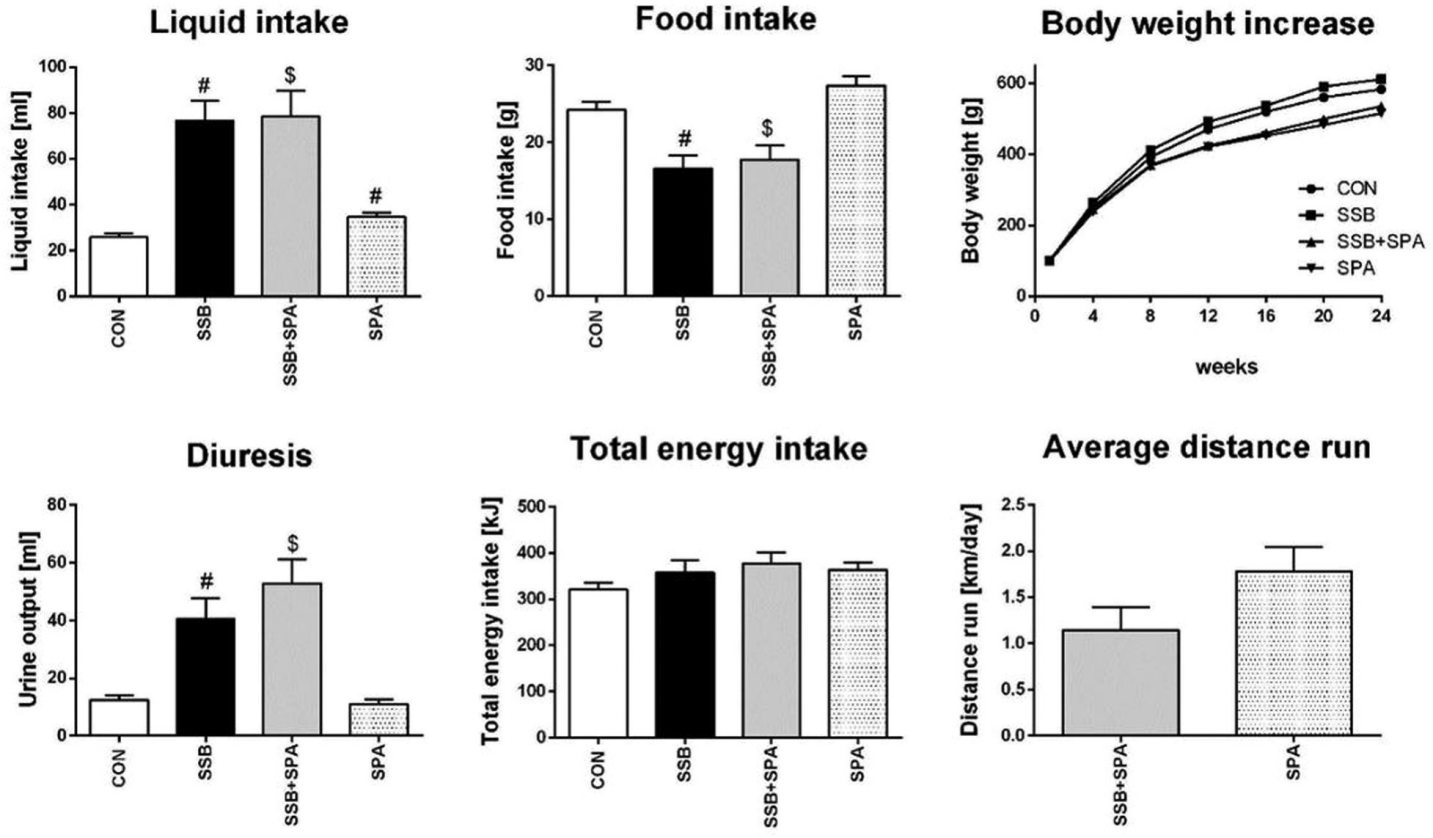
Effect of SPA on fluids and pellets intake and diuresis at the end of follow-up in control and cola SBP drinking rats and on the long-term body weight gain during follow-up and daily average distance ran. CON = control group, SSB = group drinking sugar sweetened beverage, SSB + SPA = group drinking sugar sweetened beverage concomitant to spontaneous physical activity, SPA = group performing spontaneous physical activity. Average ± SEM. *n* = 10 per group, *n* = 6–7 in daily distance run figure; ^#^ *p* < 0.01 vs CON, ^$^ *p* < 0.01 vs SPA.

**Figure 3:**
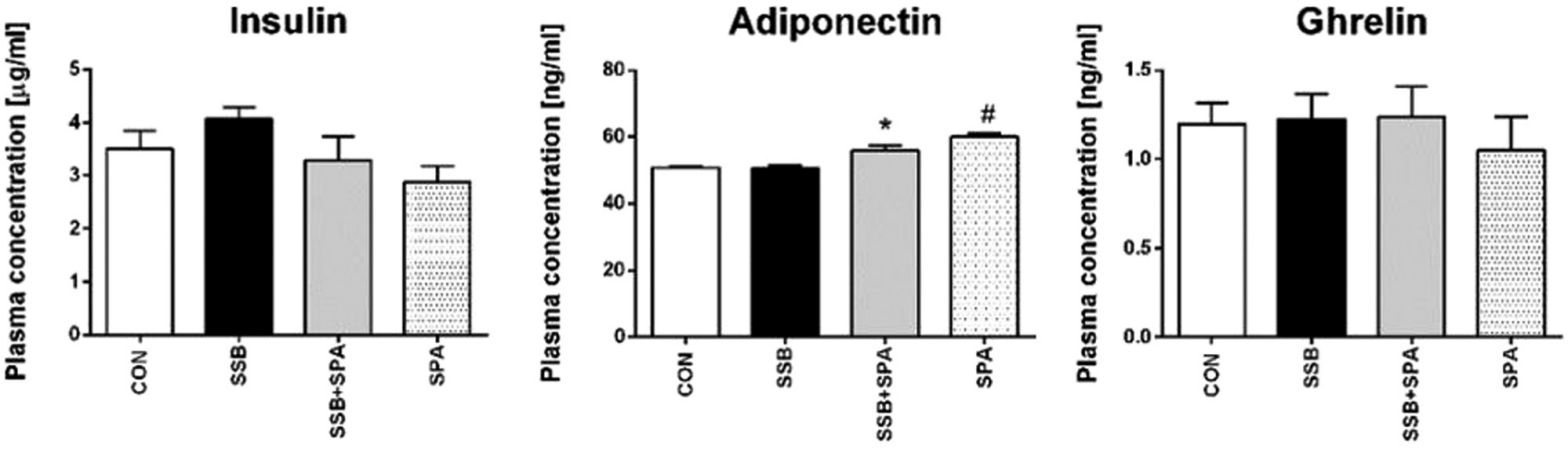
Effect of SPA on insulin, adiponectin, and ghrelin levels in plasma of controls and cola SSB drinking rats. CON = control group, SSB = group drinking sugar sweetened beverage, SSB + SPA = group drinking sugar sweetened beverage concomitant to spontaneous physical activity, SPA = group performing spontaneous physical activity. Average ± SEM. *n* = 8 per group; *p < 0.05 vs SSB, ^#^ *p* < 0.05 vs CON.

Long-term consumption of sweetened cola beverages led to only an insignificant increase in body weight in young rats (Table 2; Figure 2). On the other hand, voluntary physical activity suppressed a body weight increase in both the sweetened beverage drinking group (SSB + SPA) and the control group (SPA) during 6 months duration of the experiment. Sweetened cola beverage intake increased by 41%, although non-significant, in retroperitoneal white adipose tissue mass and non-significant increase in TAG plasma concentration by 20% in the sugar-sweetened beverage drinking group. On the other hand, physical activity significantly reduced weight gain, white adipose tissue mass, and plasma TAG levels both in control and sugar-sweetened beverage drinking rats (Table 2). Following cola beverage feeding, random as well as fasting glycemia remained stable, suggesting effective compensatory mechanisms in young rats. This was in line with only slightly increased (by 16%, Figure 3) insulin plasma levels in sedentary animals. Physical activity did not influence glycemia-related features after sugar-sweetened beverage intake but led to decreased fasting glycemia levels (by 18%, *p* < 0.05).

**Table 2:** Effect of SPA on body weight and weight gain related parameters

	CON	SSB	SSB + SPA	SPA
Final body weight (BW) (g)	574.5 ± 12.4	615.6 ± 29.4	534.8 ± 16.8^*^	513.4 ± 15.5^#^
BW increase (%)	491.9 ± 23.0	514.6 ± 27.9	443.1 ± 31.5^*^	413.6 ± 37.9^#^
WAT weight (g)	6.4 ± 0.3	9.0 ± 1.4	5.4 ± 0.6^*$^	3.9 ± 0.4^#^
Liver weight (g)	17.0 ± 0.6	21.5 ± 1.5	18.2 ± 0.7^*^	16.2 ± 0.9
Plasma TAG concentration (mmol/L)	2.5 ± 0.2	3.0 ± 0.5	1.9 ± 0.2^*^	1.5 ± 0.1^#^
Fasting glycemia (mmol/L)	6.59 ± 0.24	6.30 ± 0.26	5.81 ± 0.29	5.40 ± 0.38^#^
Random glycemia (mmol/L)	6.84 ± 0.21	6.77 ± 0.64	6.84 ± 0.43	7.07 ± 0.32

CON, control group; SSB, group drinking sugar sweetened beverage; SSB + SPA, group drinking sugar sweetened beverage concomitant to spontaneous physical activity; SPA, group performing spontaneous physical activity; WAT, white adipose tissue; TAG, triglycerides; AUC, area under the curve in oral glucose tolerance test. Number of animals = 10 per group, average ± SEM, *p < 0.05 vs SSB, ^#^ *p* < 0.05 vs CON, ^$^ *p* < 0.05 vs SPA.

### 3.3 Effect of physical activity on energy intake

Cola beverage drinking rats significantly increased their liquid intake (by approximately 200%, *p* < 0.05) but compensated for this supply of fluid-based sugars by a significant reduction of pellets intake (by approximately 31%, *p* < 0.05). This was observed independently of exercise or sedentary settings. Interestingly, compensatory reduction of chow intake resulted in similar energy intake per 24 h in all rat groups (Figure 2).

### 3.4 Effect of physical activity on inflammatory mediators in plasma and white adipose tissue

**Figure 4:**
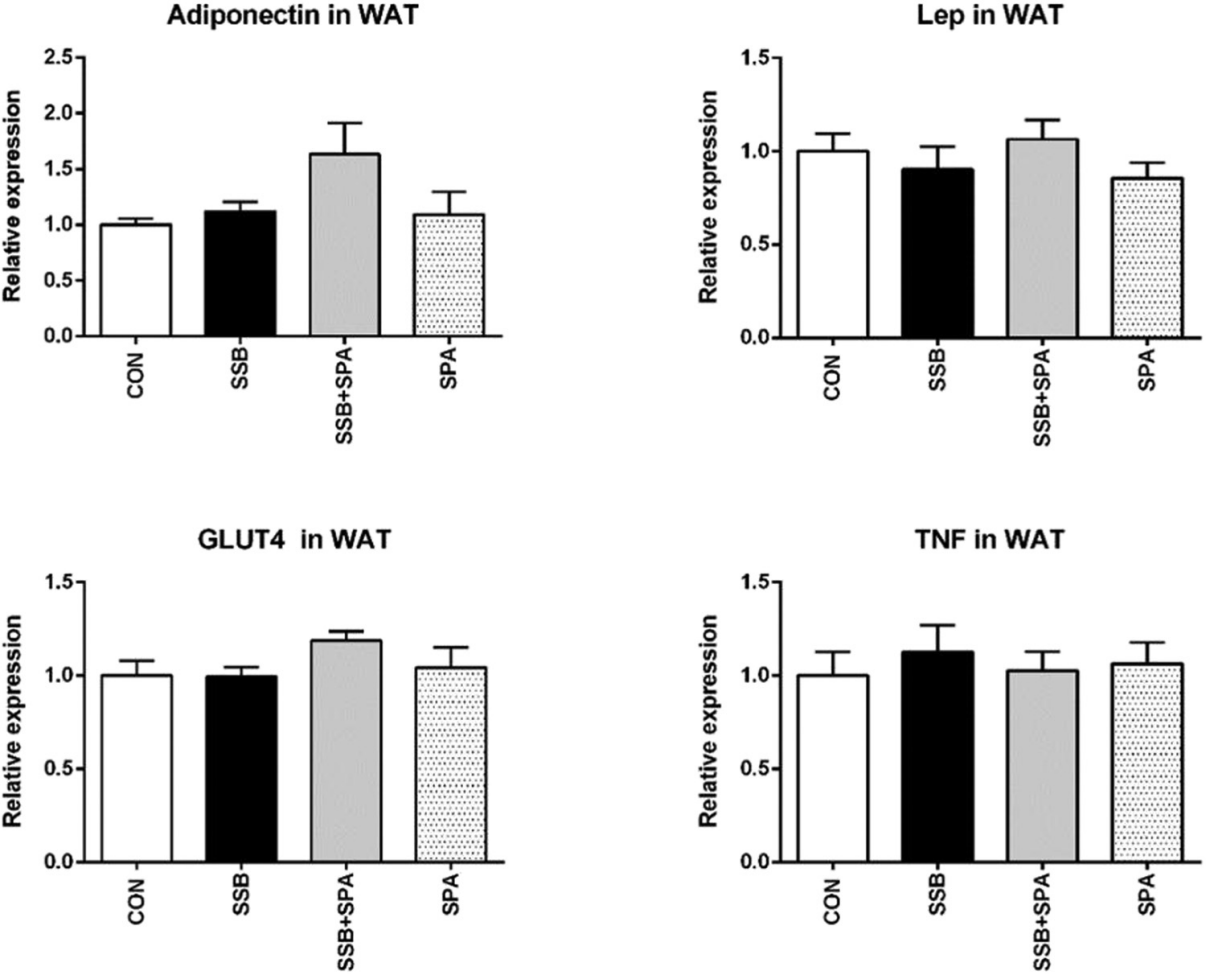
Effect of SPA on relative mRNA expression of TNF, Lep, and GLUT4 in white adipose tissue (WAT) of control and cola drinking rats. CON = control group, SSB = group drinking sugar sweetened beverage, SSB + SPA = group drinking sugar sweetened beverage concomitant to spontaneous physical activity, SPA = group performing spontaneous physical activity. Average ± SEM. *n* = 10 per group; *p < 0.05 vs SSB.

In contrast to insulin and ghrelin, which remained stable under all studied conditions, plasma levels of adiponectin were significantly elevated in both activity groups (Figure 3), which possibly mirrors the decreased proportion of adipose tissue in active rats when compared to sedentary controls. Interestingly, mRNA levels of adiponectin in white adipose tissue were significantly increased only in the group drinking sugar-sweetened beverage while performing physical activity (SSB + SPA) (Figure 4).

Exclusively, the glucose transporter type 4 (GLUT4) expression was elevated in white adipose tissue of rats drinking sugar-sweetened beverages while performing physical activity (Figure 4), suggesting a better insulin-related glucose utilization in fat tissue following activity in rats drinking cola beverage. Other indicators of altered activity of adipose tissue, metabolic [leptin (Lep)], or inflammatory [tumor necrosis factor (TNF)] indicators remained unaltered, suggesting that although the proportion of adipose tissue in the whole body increased, its function remained in a normal, probably healthy mode.

## 4 Discussion

In this study, we investigated whether physical activity influences weight gain and related biochemical and molecular indicators in settings of chronic unhealthy feeding. The main finding was the influence of exercise on fecal lactobacilli abundance in rats during a long-term administration of sweetened cola beverage in early adulthood.

Physical activity per se was insufficient to alter lactobacilli abundance in our study. This is in contrast to a study in mice, where voluntary wheel running was found to alter gut microbiota composition by increasing the number of *Lactobacillus* spp. compared to sedentary mice [22] and also to the current view that physical (in)activity can lead to a gut microbiota change [28]. On the other hand, also in humans, a causal relationship between exercise and gut microbiota composition has not been yet fully established and is thought to be directly linked with dietary adjustments [29]. Thus, it is conceivable that a combination of exercise with diet is required for microbiota change.

In line with this hypothesis, we documented a rise of lactobacilli in the group drinking sugar-sweetened beverages while performing physical activity. It has been previously observed in similar animal experiments that fecal samples of obese rats were enriched with *Lactobacillus* spp., specifically after exercise [16]. In general, numerous strains of lactobacilli may positively influence not only gastrointestinal health but also performance in athletes [25,26]. However, the mechanism by which microbiota affects exercise and vice versa has not been supported by substantial evidence. Alteration of gut motility is one mechanism by which exercise may influence the microbiome [30]. Exercise can also reduce intestinal blood flow [31], which could potentially lead to reduced absorption of fructose from the gut, thus increasing the availability of the sugars to the microbes. It has been recently reported that dietary fructose is converted by the microbiome into short-chain fatty acids, such as acetate [2], and acetate is most likely to be an important energy source in the skeletal muscle, especially during endurance exercise [32]. Taken together, this would suggest that exercise could lead to increased production of acetate from dietary fructose, which would potentially, in turn, further increase exercise endurance.

Interestingly, in our experiment, rats drinking sugar-sweetened beverages did not exhibit increased exercise performance compared to rats performing physical activity without access to such drink (rather non-significantly decreased). From our perspective, the lack of enhanced performance in the exercising group while having access to cola-sweetened beverages is somewhat unexpected, but it is suggestive that the compensatory reduction of feed may influence effective energy production. Another explanation may simply lie in the fact that sugar-sweetened beverage drinking rats, though exercising, still had significantly larger adipose tissue mass compared to control exercising animals, which could be limiting the activity.

As mentioned above, when comparing a total caloric intake by calculating the sum of energy from liquid and food ingested, we discovered that decreased food intake in both sweetened beverage drinking groups contributed to total energy intake comparable to the control group. This remained similar also under increased energy expenditure conditions in exercising animals. Indeed, one of the typical phenomena observed in animal experiments with sugar-sweetened beverage administration is the compensatory reduction of feeding [33]. It is thought to be a result of drinking large volumes of high caloric drinks, such as cola, which provides excess caloric intake [34], or could be connected to a high content of caffeine in cola beverages, which is known for its direct appetite suppressing effect [35,36]. Nevertheless, in spite of unchanged total caloric intake, favoring the high fructose intake may directly enhance body adiposity and microbiota composition.

We have observed an increase in adiponectin plasma levels in both exercising groups. However, only a combination of exercise and sweetened beverage intake also exhibited an increase in mRNA levels of adiponectin in white adipose tissue. Adiponectin is a circulating protein with a role in metabolism regulation and inflammation and is produced by more tissues, including the white adipose tissue and skeletal muscle [37]. Even though lower adiponectin levels are often observed in obese [38] and diabetic populations [39], exercise can restore plasma adiponectin concentration. This is metabolically relevant due to adiponectin’s insulin-sensitizing, anti-inflammatory, and antioxidant properties [38]. Similar to our results, it was previously reported that endurance training leads to increased adiponectin mRNA levels in mice fed a high-fat diet [40]. Our results suggest that exercise can improve adiponectin plasma concentration within both normal as well as unhealthy feeding. In the case of sugar-sweetened beverage intake, this effect is apparently related to alterations in the white adipose tissue.

There are certain limitations in our study that need to be considered. We measured only the white adipose tissue mRNA expressions, while methods determining protein levels would provide more detailed information. The gut motility, as well as plasma acetate concentration, was not monitored. Although not the aim of this study, changes in other bacterial genus may provide a deeper understanding of interactions between microbiota and exercise.

## 5 Conclusion

Our results demonstrate that spontaneous physical activity leads to the prevention of weight gain and improvement of lipid-related features of obesity and increases the plasma levels of adiponectin. It can also be concluded that spontaneous physical activity in young rats drinking sweetened cola is accompanied by increased abundance of lactobacilli in the fecal microbiota population. This suggests that lactobacilli may require an additional source of energy in the form of sugars present in the sugar-sweetened beverage used in this experiment: gut microbiota is known to metabolize fructose into short-chain fatty acids, which can serve as a source of energy for a skeletal muscle. This proposes a research area of interest – can lactobacilli in connection with exercise be directly involved in obesity prevention?
